# Attachment-Based Parenting Interventions and Evidence of Changes in Toddler Attachment Patterns: An Overview

**DOI:** 10.1007/s10567-022-00405-4

**Published:** 2022-08-18

**Authors:** Jane Kohlhoff, Corey Lieneman, Sara Cibralic, Nicole Traynor, Cheryl B. McNeil

**Affiliations:** 1 School of Psychiatry, University of New South Wales, PO Box 241, Villawood, NSW 2163 Australia; 2 Karitane, Villawood, Australia; 3 Munroe-Meyer Institute, University of Nebraska Medical Center, Omaha, Australia; 4 Department of Psychology, West Virginia University, Morgantown, USA

**Keywords:** Attachment, Toddler, Parenting, Attachment-based interventions

## Abstract

There is strong evidence to show links between attachment security in young children and a range of positive outcomes in social, emotional, and psychological domains. The aims of this review were to provide a narrative summary of (1) the attachment-based interventions currently available for caregivers of toddlers aged 12–24 months and for which research about the impact of the program on child attachment patterns has been reported, and (2) the empirical effectiveness of these interventions at improving attachment security. A number of interventions were shown to be associated with shifts to secure and/or organized attachment, with Child-Parent Psychotherapy and Attachment and Biobehavioral Catch-Up emerging as the interventions with the strongest evidence bases. For most interventions, evidence came from just a single research study, and in some cases from studies that were not randomized controlled trials. In order for clinicians to make informed decisions about the interventions they use with parents and toddlers, it is vital that further research be conducted to test the efficacy of all available attachment-based parenting programs using randomized controlled trial designs, in a range of settings and clinical and cultural groups, and with longitudinal follow-ups.

There is strong evidence to show that the quality of parenting that a child receives during the earliest years of life can have a significant impact on the developing parent–child attachment relationship (Wolff & Ijzendoorn, 1997), and on the child’s social, emotional, and psychological outcomes (Moore et al., 2017). With this in mind, various attachment-based parenting interventions have been developed with the aim of enhancing parenting quality and promoting parent–child attachment security in the early years (Steele & Steele, 2018). The current paper seeks to provide an overview of the attachment-based parenting interventions that are currently available for caregivers of toddlers aged 12–24 months, along with an overview of the empirical effectiveness of these interventions at improving attachment security in children of this age group.

## Toddlerhood

The early toddler years, the period of life from around 12 to 24 months, represent a crucial developmental phase. During this time, children undergo significant and rapid growth in a range of areas including physical mobility, capacity for language and social relationships, play, independence and sense of self-identity, cognitive abilities, and capacities for self-regulation and emotional regulation (Crockenberg & Leerkes, 1993; Lieberman, 1993; Sroufe, 1995; Woody, 2003). With heightened levels of neuroplasticity and susceptibility to the influences of the environment, the quality of early parenting during this phase of life plays a key role in shaping the developmental and mental health trajectories (Lomanowska et al., 2017; Moore et al., 2017). Evidence suggests that children who experience adverse early caregiving (e.g., non-sensitive, harsh, hostile, or inconsistent parenting) in the toddler years are at elevated risk of a range of concurrent and subsequent social–emotional and behavioral issues (Mendez et al., 2016; Samdan et al., 2020; Van Aken et al., 2007; Wiggins et al., 2015), which in turn, place them on pathways to poor mental health throughout childhood, adolescence, and beyond (Campbell et al., 2006; Kim-Cohen et al., 2003).

## Attachment Theory

Attachment theory is an ethological theory that seeks to account for the impact of the early parenting environment on a child’s social–emotional development and behavior (Bowlby, 1988). Originally proposed by Bowlby (1969) and further developed in the work of Ainsworth (Ainsworth et al., 1978), attachment theory argues that social, emotional, and cognitive capacities develop and flourish from infancy through the early toddler years in the context of early caregiving that is sensitive and contingently responsive (Lyons-Ruth, 1996), or in other words, caregiving that involves accurately perceiving and interpreting a child’s signals followed by prompt, appropriate responding (Ainsworth et al., 1978). Specifically, attachment theory suggests that it is through repeated experiences of sensitive and responsive interactions with the caregiver that the infant or young toddler develops an internal working model of the primary caregiver as a “secure base” from which he or she can explore the environment, and feel assured that comfort and protection will be available when required. Conversely, insecure attachment patterns develop when the child experiences repeated interactions with a caregiver in which his or her bids for parental proximity/emotional support are rejected or met inconsistently. According to attachment theory, children who have experienced this type of caregiving typically develop internal working models of the parent as unavailable or unreliable, and thus they engage in compensatory strategies (attachment avoidance or attachment ambivalence/resistance) to deal with the resulting relational stress. In addition, infants and toddlers who experience abusive, frightening or frightened parenting may fail to develop a coherent or organized attachment strategy. These children typically show odd, disorganized behaviors in the presence of key attachment figures (Main & Solomon, 1986, 1990).

The two most established methods for assessing attachment in young children are the strange situation procedure (SSP) (Ainsworth et al., 1978) and the Attachment Q-Set (AQS) (Waters, 1995). The SSP is a standardized, lab-based procedure that comprises a series of 8, 3-min episodes involving separations and reunions between a child (aged 12–18 months), his or her parent, and a ‘friendly’ stranger. Child behaviors in response to separations and reunions with the parent are observed and used to inform the allocation of 3-way and 4-way attachment classifications (Ainsworth et al., 1978). For the 3-way classification system, a primary classification of ‘secure,’ ‘insecure-avoidant,’ or ‘insecure-ambivalent/resistant’ is given (with disorganized attachment forced into one of the three organized categories). For the 4-way classification, a primary classification of ‘secure,’ ‘insecure-avoidant,’ ‘insecure-ambivalent/resistant,’ or ‘disorganized’ is given, with the disorganization classification allocated for children who are observed to lack a coherent strategy of responding to distress associated with the caregiver, e.g., odd, disoriented behaviors; (Main & Solomon, 1986, 1990). As an alternative to the SSP, the AQS (Waters, 1995) was developed as a 90-item observer Q-sort procedure for use in naturalistic settings. The AQS was designed for use with children aged 12–48 months and is typically scored from a 60–90-min parent–child observation in the family home. It yields a continuous attachment security score ranging from − 1.00 (least like a securely attached child) to + 1.00 (most like a securely attached child).

The empirical evidence to support attachment theory is strong. Meta-analytic studies have identified maternal sensitivity as a significant predictor of infant attachment security (Wolff & Ijzendoorn, 1997). Numerous studies have documented links between insecure or disorganized attachment measured in infancy and a range of compromised outcomes later in life including externalizing and internalizing behaviors and self-regulation difficulties in middle childhood (Boldt et al., 2020; Fearon et al., 2010; Madigan et al., 2013), and social, emotional, and mental health difficulties in adolescence and adulthood (Carlson, 1998; Girme et al., 2020). Importantly, attachment security has been shown to be protective for children impacted by known risk factors such as poverty (Delker et al., 2018) and parental substance abuse (Edwards et al., 2006).

## Attachment-Based Parenting Interventions

In response to the identified links between early parenting quality and infant/toddler attachment security, and between infant/toddler attachment security and subsequent positive social, emotional, and psychological outcomes, attachment-based parenting interventions have been developed and recommended (Moore et al., 2017; Steele & Steele, 2018). Attachment-based interventions aim to improve parental capacity to provide sensitive and responsive caregiving, with the ultimate goal of improving child attachment patterns. While these interventions have a common goal, the methods and foci of individual interventions vary, with some programs intervening at a behavioral level (e.g., using live coaching, using video feedback with parents to target specific parental behaviors), and others focus on changing caregiver representations (e.g., parental reflective functioning; Wolff & Ijzendoorn, 1997).

## Aims of the Current Paper

Given the growing empirical evidence base for the importance of the toddler years and the key role of early parenting and the parent–child attachment relationship in shaping child outcomes, many countries have started to develop health strategies and policies focusing on supporting early parenting, in the hope that the significant social and economic burdens presented by adult mental illness will be reduced (Australian Government National Mental Health Commission, 2021; Australian Government Productivity Commission, 2021). In this context, it is vital that clinicians who are working with young toddlers and families maintain a clear understanding of both the available attachment-based interventions, and also the research evidence to support these interventions. In particular, it is important that clinicians are aware of the ability for these interventions to bring changes in parent–child attachment relationships. To this end, the current paper aims to provide a narrative summary of (1) the attachment-based interventions currently available for caregivers of toddlers aged 12–24 months, and (2) the empirical effectiveness at improving attachment security.

## Method

A literature search was conducted using PsycINFO and PsychARTICLES through June of 2020 to identify relevant articles. Search terms included attachment, attachment intervention, treatment, pre-, post-, measures, SSP, 12–24 months, toddlers, Circle of Security, COS, Attachment and Biobehavioral Catch-Up, ABC, Child-Parent Psychotherapy, and CPP. Reference lists of meta-analyses on this topic were also searched manually. Attachment studies in which no intervention occurred, interventions occurred only outside of our 12–24-month age range, or no attachment outcomes were measured, were excluded. Theoretical articles, commentaries, dissertations, theses, poster presentations, case studies, and publications not available in English were also excluded.

## Results

Ultimately, 22 articles were selected for inclusion in this review. The following section provides a description of the attachment-based interventions for caregivers of toddlers aged 12–24 months, and available attachment outcome data; a summary is provided in Table 1.

**Table 1:** Overview of parenting interventions and evidence regarding changes in infant attachment

Intervention	Outcome study author(s)	Study design	Sample size	Child age (months) at program commencement	Time (s) of attachment assessment	Attachment measures used	Changes in attachment (intervention group)
Attachment and Biobehavioural Catch-Up (ABC)	Dozier et al. (2009)	RCT	*N* = 46	*M* = 18.9; *SD* = not available; range = 3.9–39.4	Pre- and post-intervention	Foster carer report using attachment diaries	Decreased avoidance
	Bernard et al. (2012)	RCT	*N* = 120	*M* = 10.1; *SD* = 6.0; range 1.7–21.4	Post-intervention	SSP	Decreased disorganization; Increased security for those at risk of maltreatment
	Zajac et al. (2020)	RCT (follow-up of Bernard et al., 2012)	*N* = 100	Same sample as Bernard et al. (2012)	9 years of post-intervention	Kerns attachment scale	Increased security
Circle of Security-Intensive (COS-I)	Hoffman et al. (2006)	Open trial	*N* = 65	*M* = 32.0; *SD* = 12.6; range 11–58	Pre- and post-intervention	SSP	Increased security; decreased combined disorganized-controlling attachment/insecure-other
	Huber et al. (2015)	Open trial	*N* = 83	*M* = 47.80; *SD* = 17.48, range 13–88	Pre- and post-intervention	SSP	No statistically significant differences in % insecure or disorganized
Circle of Security Perinatal (COS Perinatal)	Cassidy et al. (2010)	Open trial	*N* = 20	During pregnancy^a^	Post-intervention	SSP	Increased security and decreased disorganization compared to high-risk samples; no difference in rate of security or disorganization compared to a low-risk sample
COS-Home-Visiting-4 (COS-HV4)	Cassidy et al. (2011)	RCT	*N* = 220	6.5 months^a^	Post-intervention	SSP	Increased security among highly irritable infants
Child-Parent Psychotherapy (CPP)	Lieberman et al. (1991)	RCT	*N* = 93	Range = 11–14^a^	Pre- and post-intervention	SSP; AQS	Decreased avoidance, resistance, and anger; Increased partnership with mother
	Cicchetti et al. (1999)	RCT	*N* = 63	*M* = 20.40; *SD* = 2.38, range not provided	Post-intervention	AQS	Increased security
	Toth et al. (2006)	RCT	*N* = 130	*M* = 20.34, *SD* = 2.5; range not provided	Post-intervention	SSP	Increased security
Infant-Parent Psychotherapy (IPP)	Cicchetti et al. (2006)	RCT	*N* = 189	*M* = 12.31, *SD* = .81	Pre-treatment; post-intervention	SSP	Increased security; Decreased disorganization
	Stronach et al. (2013)	RCT	*N* = 189	Follow-up of Cicchetti et al. (2006)	One-year post-intervention	SSP	Increased security; Decreased disorganization
Steps Toward Effective and Enjoyable Parenting (STEEP)	Suess et al. (2016)	Quasi-experimental	*N* = 107	Birth–2 years^a^	Post-intervention & 1-year post-intervention	SSP	No statistically significant differences in % insecure or disorganized; Decreased continuous 9-point disorganization rating
Early Head Start (EHS)	Roggman et al. (2009)	RCT	*N* = 160	Pregnancy–10 months postpartum^a^	When children were 14 and 18 months	AQS	Increased security
Watch, Wait, and Wonder (WWW)	Cohen et al. (1999)	RCT	*N* = 67	10–30 months (WWW group: *M* = 21.5, *SD* = 6.5; Control group: *M* = 19.2, *SD* = 6.1)	Post-intervention & 6-month post-intervention	SSP	Increased security
UCLA Family Development Project	Heinicke et al. (1999)	RCT	*N* = 64	Late pregnancy^a^	Post-intervention	SSP	Increased security
Right From the Start (RFTS)	Niccols (2008)	RCT	*N* = 76	M = 8.4; *SD* = 5.4; range not provided	Post-intervention; 6-month follow-up	AQS	No statistically significant differences in security
Promoting First Relationships (PFR)	Spieker et al. (2012)	RCT	*N* = 210	*M* = 17.96; *SD* = 4.97; range not provided	Post-intervention & 6-month post-intervention	Toddler Attachment Sort-45	Increased attachment-related outcomes
Moss’s Home-Visiting Intervention	Moss et al. (2011)	RCT	*N* = 67	*M* = 40.2; *SD* = 1.38; range 12–71	Post-intervention	SSP; Preschool Separation–Reunion Procedure	Increased security; Increased organization
Lyons-Ruth’s Home-Visiting Intervention	Lyons‐Ruth et al. (1990)	RCT	*N* = 31	*M* = 4.7^a^	Post-intervention	SSP	Decreased insecurity; decreased disorganization
Minding the Baby	Slade et al. (2020)	RCT	*N* = 156	Pregnancy^a^	Post-intervention	SSP	Increased security; decreased disorganization
Parent–Child Interaction Therapy–Toddlers (PCIT-T)	Kohlhoff et al. (2020)	Open trial	*N* = 16	*M* = 19.02; *SD* = 2.34; range not provided	Pre-intervention; Post-intervention	SSP	No statistically significant differences in % insecure or disorganized

*RCT* Randomized controlled trial, *SSP* strange situation procedure, *AQS* Attachment Q-set

^a^Mean, SD or range not provided

### Attachment and Biobehavioral Catch-Up (ABC)

#### Intervention Overview

Attachment and Biobehavioral Catch-Up (ABC) (Bernard et al., 2012; Dozier & Bernard, 2017; Dozier et al., 2009) is a manualized, in-home intervention comprising approximately 10, 1-h sessions for families of children ages 6 to 24 months. The program aims to increase parental nurturance in response to child distress, to increase parental sensitivity and positive regard, and to decrease frightening and intrusive parental behavior. During each session, caregivers practice new skills in conjunction with “in-the-moment” therapist coaching focusing on describing child and caregiver behaviors, labeling intervention targets used, and highlighting outcomes. Sessions are carried out in the family home, and so other family members are also encouraged to join in. An adapted version of ABC for toddlers aged 24–48 months has also been described (ABC-Toddler; ABC-T) (Lind et al., 2017), emphasizing supporting children’s developing emotion regulation abilities through availability and co-regulation when toddlers are overwhelmed by their feelings. In ABC-T, caregivers are coached to remain calm, validate their toddlers’ feelings, and identify past experiences in their own lives that may be contributing to their reactions to their children’s feelings.

ABC facilitator training is provided through the University of Delaware (University of Delaware, 2017). Training involves a 2-day training course, followed by 12 months of supervision (2 supervision meetings per week).

#### Evidence Base

Three studies have investigated changes in child attachment following ABC (Bernard et al., 2012; Dozier et al., 2009; Zajac et al., 2020). In an early investigation, Dozier et al. (2009) used a randomized control trial (RCT) to assess the effectiveness of ABC in improving avoidant attachment compared to a control condition (the Developmental Education for Families intervention; Brooks-Gunn et al., 1993; Ramey et al., 1982) when delivered to foster carers; attachment was assessed using attachment diaries completed by the foster carers. The sample comprised 46 children (child age range = 3.9–39.4; *M* = 18.9 months) and their foster carers (91% female). Results showed that children whose foster carers had received ABC reported less avoidant attachment behaviors in the child compared to those who received the control intervention. Study limitations included the small sample and reliance on a non-gold standard caregiver-report measure of attachment.

Bernard et al. (2012) conducted a RCT to evaluate the efficacy of ABC compared to a control treatment (the Developmental Education for Families intervention) in improving attachment (assessed using the SSP) in a sample of 120 parent–child dyads where the children were at risk of maltreatment (child age range = 1.7–21.4; *M* = 10.1, *SD* = 6.0 months; parents 98% female). Their results indicated that children in the ABC treatment group showed significantly lower rates of disorganized attachment and higher rates of secure attachment at post-treatment compared to those in the control treatment group. Although treatment groups were randomized, this study was limited by the fact that it did not assess baseline attachment security. Given that the children in the study varied in age at the point of recruitment (on average they were aged 6 months, with some as young as 1.7 months), assessing baseline attachment was obviously not possible. It does, however, limit the interpretability of results as it is not possible to know whether the groups differed in terms of child attachment, prior to receiving the intervention. Zajac et al. (2020) followed up the children in the Bernard et al. (2012) sample when they were 9 years of age (*N* = 100; ABC group *n* = 44, *Mage* = 9.45, *SD* = 0.34 years; control group *n* = 56, *Mage* = 9.46, *SD* = 0.38 years). Results showed that children whose parents had received ABC reported higher levels of attachment security on a self-report scale (Kerns Security Scale) (Kerns et al., 1996) than children whose parents had received the control intervention (Zajac et al., 2020). While the authors found no statistically significant demographic differences between participants who completed the 9-year follow-up and those who were enrolled but who dropped out, the high level of attrition (*n* = 100 children completed the 9-year assessment, from a total sample of *n* = 212 originally enrolled in the intervention) represents a significant study weakness. The reliance on a self-report measure of attachment was a further limitation. To date, to our knowledge, no empirical research investigating attachment outcomes of ABC-T have been published.

### Circle of Security (COS)

#### Intervention Overview

The COS intervention model seeks to improve children’s attachment outcomes by increasing caregiver sensitivity and empathy, improving caregiver attunement to children’s attachment-related cues, understanding how caregivers’ past experiences influence current parenting, and using groups as a secure base from which to explore relationships. The original COS program, now referred to as Circle of Security-Intensive (COS-I; Powell et al., 2014), is a 20-week group intervention in which groups of five to six caregivers and a therapist meet for about 75 min each week. Caregivers interact with their children in supplemental behavioral observations, which are video-taped. To individualize treatment, COS-I uses baseline assessments of caregiver and child attachment-related variables as well as video clips of group members’ child–caregiver interactions.

Several adaptations of COS-I have been developed, the most well-known being COS-Parenting (COS-P; Cooper et al., 2009), a 10-week intervention designed for broader implementation. COS-P includes many components of the original COS-I intervention but focuses more on education, using pre-recorded videos of caregiver–child interactions instead of videos of group participants. Other adaptations of the original COS-I protocol include COS Perinatal Protocol, developed for delivery to parents during pregnancy up to 12 months of postpartum (Cassidy et al., 2010); COS-Home-Visiting-4 (COS-HV4), a home-based protocol that includes video review of infant–child interactions as well as live feedback to caregivers during home-based parent–infant interactions (Cassidy et al., 2011); COS hybrid model, which comprises both the Circle of Security Parenting material and individualized video reviews (Huber et al., 2020); and Circle of Security in the Classroom, developed for childcare and preschool teachers (Cooper et al., 2017; Gray, 2015).

COS facilitator training is delivered in multiple locations worldwide, through Circle of Security International (Circle of Security International, 2019). Training for COS-P involves a 2-day training course. Registered COS-P facilitators can then undertake COS-I training comprising a 5-day training course followed by self-guided video-based case reviews and a period of supervision around COS-I cases.

#### Evidence Base

Two studies have examined attachment outcomes following COS-I, both of which also included children aged older than 24 months (Hoffman et al., 2006; Huber et al., 2015). In the earliest study, Hoffman et al. (2006) assessed infant attachment (using the SSP) in a sample of 65 caregiver–child dyads recruited from Head Start programs (child age range = 11–58, *M* = 32.0, *SD* = 12.6 months), pre- and post-COS-I. Of the 65 caregivers, most were mothers (86%) and the others were foster carers (6%), fathers (6%), and grandparents (2%). Results showed that the rate of secure attachment improved from 20% at pre-treatment to 54% at post-treatment, and showed decreases in the combined rates of disorganized-controlling attachment and insecure-other attachment from 60% at pre-treatment to 25% at post-treatment. These results suggest that the program is associated with significant attachment changes for many families, but the lack of a control group means that results should be interpreted with caution. In a subsequent Australian study, Huber et al. (2015) employed an open-trial design to investigate changes in attachment security (assessed using the SSP) following the COS-I intervention delivered to a sample of 83 carers including biological parents (88%), foster/adoptive parents (6%), and kinship carers (6%) (child age range = 13–88, *M* = 47.80, *SD* = 17.48 months). Results showed no significant differences from pre- to post-treatment in the percentages of children classified as secure or disorganized. The authors noted, however, that at the post-treatment assessment, participating children showed significantly more secure attachment behaviors and fewer disorganized attachment behaviors than they had done at the pre-treatment assessment. Due to a large attrition rate (44% drop outs/missing post-treatment data) the authors re-analyzed the data using an intention-to-treat design, but results were unchanged. As was the case for the Hoffman et al. (2006) study, the lack of a control group in this study was a significant limitation.

Attachment outcomes following a number of the adapted versions of COS have been tested. Cassidy et al. (2010) investigated attachment outcomes of the COS Perinatal Protocol in a sample of 20 mothers and infants as part of a larger intervention project in which the COS Perinatal was employed as part of a 15-month residential treatment program for pregnant women with histories of substance abuse who had been convicted of non-violent crimes (Cassidy et al., 2010). Results showed that at post-treatment, participating children demonstrated rates of attachment security and organization (assessed using the SSP) comparable to those of children from low-risk samples. However, the 20 study participants represented only 55% of the original sample, while treatment completers and non-completers were found to be comparable on a number of demographic and psychological variables, the study completers were on average, more educated. The other major study limitation was the fact that there was no control group. In 2011, Cassidy et al. (2011) employed an RCT to compared outcomes of COS-HV4 versus a psycho-educational control intervention in a sample of 220 mother–child dyads (enrolled in the study when infants were 1 month of age). There was a high level of participant retention (94%) and results showed that highly irritable infants in the treatment group showed significant improvements in attachment security (assessed using the SSP). The study was conducted with an economically stressed sample of mothers with irritable infants, so an important future research direction would to be replicate results in other populations. While one RCT has examined attachment outcomes in a pre-school age sample following COS-P (Cassidy et al., 2017), no studies have investigated attachment outcomes following the COS-P intervention in children aged 12–24 months.

### Child-Parent Psychotherapy (CPP)

#### Intervention Overview

Child-Parent Psychotherapy (CPP) is an attachment-focused dyadic child–caregiver intervention for children ages zero to five years who have experienced trauma (Lieberman, 2004). Other versions of CPP have included Infant-Parent Psychotherapy (IPP) (Lieberman & Pawl, 1993) and Toddler-Parent Psychotherapy (TPP) (Lieberman, 1992). In CPP, families typically receive 60–90-min unstructured weekly visits to the home for one year from a therapist. The intervention aims to respond to emotional experiences between mother and child, including addressing the mother’s concerns about psychological conflict with her child, and to provide appropriate developmental information tailored to the child. CPP focuses on understanding the ways in which past trauma and history of the child–caregiver relationship impact current child–caregiver functioning with emphasis on caregivers’ and children’s mental representations of the self and the other. The goals of CPP are to improve caregiver responsiveness to child affective communication, return the child to a healthy developmental trajectory, improve the child–caregiver relationship, and construct a joint trauma narrative.

Training in the CPP model is provided by endorsed CPP trainers listed on the Child Trauma Research Program CPP website/Trainer Roster (University of California San Francisco, 2022). CPP implementation training takes place through three different mechanisms: CPP Learning Collaborative or Learning Community (18 months of duration), CPP Agency Mentorship Program (18 months of duration), and Endorsed CPP Internship (12 months of duration). Each mechanism involves a mix of didactic training, learning through clinical practice and case presentations, supervision/consultation, and agency/team support. Training participants are eligible for the CPP roster when they have completed the minimum CPP training requirements (Child-Parent Psychotherapy, 2022).

#### Evidence Base

In an early study, Lieberman et al. (1991) studied 93 mother–child dyads (child age range = 11–14 months) all of whom were recent Mexican immigrants and of low socioeconomic status. At study entry, the SSP was administered; children classified as insecurely attached were then randomly assigned to CPP (insecure CPP) or a non-treated control group (insecure controls), and securely attached children formed a second non-treated control group (secure controls). When children were followed up one year later (with an 18% attrition rate; rate did not differ between the experimental groups), children in the insecure CPP group showed less avoidance, resistance, and anger, and more partnership with their mothers compared to insecure controls. There were no significant differences between children in the insecure CPP group and secure controls. There were also no AQS security score differences between the insecure CPP and the insecure control groups.

Cicchetti et al. (1999) conducted a RCT in which 63 children (*M* age = 20.40, *SD* = 2.38 months) of depressed mothers were randomly allocated to receive TPP or to be non-treated controls (depressed controls). A third group comprised children of non-depressed mothers (*n* = 45; non-depressed controls). Child attachment security was rated using the AQS at baseline and follow-up, with a cluster analysis technique utilized to classify children as secure or insecure. At baseline, children in the TPP and depressed control groups had comparable but higher levels of attachment insecurity than children in the non-depressed control group. In contrast, at post-treatment, the TPP and non-depressed controls had comparable levels of attachment security, and children in the depressed control groups had higher levels of attachment insecurity. This study was limited by a significant rate of attrition/missing data, with only 54% of the mother–child dyads who were originally recruited having completed the post-intervention attachment measure (AQS). The authors did, however, show that participants who did and did not complete pre- and post-AQSs were largely indistinguishable, except that mothers who did not complete a post-AQS reported higher levels of stress at baseline. In a subsequent RCT, Toth et al. (2006) recruited 130 children whose mothers had experienced depression in the time since they were born, and randomly assigned them to TPP or no-treatment conditions. They also recruited 68 children whose mothers had not experienced depression in their lifetime, who formed a second control group. The children were, on average, aged 20.34 months (*SD* = 2.5). At baseline, the children of depressed mothers showed lower rates of attachment security (assessed using the SSP) than those of non-depressed mothers. In total, 82.3% of the baseline sample was re-assessed post-treatment. Results showed children who had completed TPP showed higher rates of attachment security than both the no-treatment control children and children of non-depressed mothers, as measured by the MacArthur Preschool Attachment Coding System (Cassidy et al., 1989).

Cicchetti et al. (2006) conducted a RCT to examine the impact of CPP versus a psycho-education parenting intervention (PPI) and a community control condition in a sample of 137 one-year-old children from maltreating families (child age *M* = 12.31, *SD* = 0.81 months) and their mothers. A fourth group of children from non-maltreating families was also studied as normative controls. The authors reported that a large proportion of the participants who were recruited did not go on to receive treatment (39.6% of those allocated to CPP; 51% of those allocated to PPI) but there were no significant differences between completers and drop outs in terms of demographics or baseline measures. At baseline, the children with histories of maltreatment demonstrated higher rates of disorganized attachment (assessed using the SSP) compared to children from the non-maltreated control group. One year later, at the conclusion of the treatment period (child age approximately 26 months), children in the CPP and psycho-education groups showed decreased rates of disorganized attachment and increased rates of attachment security, but children in the community control and non-maltreatment control groups showed no changes in these areas. In a subsequent study, Stronach et al. (2013) followed the same sample up 12 months after the completion of treatment (i.e., when the children were approximately 38 months of age). Overall, 78.3% of families participated in the follow-up, with no differences in the rates of participation among families in the CPP and PPI condition. Results showed that the increased rates of attachment security and decreased rates of disorganization observed at the 1-year follow-up for the CPP and psycho-education groups were sustained. These authors also found that while there were no differences in the rates of attachment disorganization between the CPP and psycho-education groups, the rate of attachment security was higher in the CPP group. While Stronach et al. (2013) results are encouraging, the study was conducted in a sample of high-risk maltreating families and so further studies in different populations are required to determine the generalizability of results to other groups.

### Steps Toward Effective and Enjoyable Parenting (STEEP)

#### Intervention Overview

Steps Toward Effective and Enjoyable Parenting (STEEP; Egeland et al., 1993) is an intervention program involving home visits and a parenting group, designed for first-time mothers at elevated risk of parenting issues due to factors such as poverty, lack of education, youth, social isolation, and adverse life circumstances (Erickson et al., 1992). Delivered from the second trimester through to the child’s first birthday, STEEP aims to increase caregivers’ sensitive responding and improve the quality of the early parent–child relationship. Key strategies include providing psycho-education about infant cues and developmental milestones, wraparound support for caregivers, and a psychodynamic approach to resolving issues around caregivers’ attachment history (Korfmacher et al., 1997). STEEP facilitators help caregivers to develop insight around their own early childhood experiences and the impact of these on current caregiving. The therapeutic relationship also serves as a corrective experience for caregivers’ insecure attachment relationships (Erickson et al., 1992).

Training in the STEEP model is provided through the University of Minnesota Center for Early Education and Development (University of Minnesota). Training involves a 2-day workshop followed by a 1-day follow-up consultation several months later.

#### Evidence Base

There is evidence of positive changes in infant attachment following STEEP delivered to parents in the first postpartum year (Heinicke et al., 1999) and one study has investigated attachment outcomes when STEEP was extended through the second year of the child’s life. In this German study, Suess et al. (2016) used a quasi-experimental design to compare attachment outcomes for 78 mother–child dyads who received STEEP compared to 29 dyads who received standard German welfare system services. Results showed that at the end of the intervention (child age 24 months), the odds of the child being classified using the SSP as insecure or disorganized were no different in the STEEP than the control group. The STEEP group did, however, show lower scores on the continuous 9-point disorganization rating scale of the SSP, when controlling for risk exposure. At this same time point, there was no significant difference between groups in terms of child attachment security as assessed using the AQS, but the authors reported that the effect was in the expected direction, with a medium effect size. Results of this study were limited by the quasi-experimental design, and the high rates of participant drop out/missing data (49.5% in the STEEP group and 51.7% in the control group).

### Early Head Start (EHS)

#### Intervention Overview

Established in 1994, EHS is a national early intervention program in the United States for low-income families from pregnancy through child age 3 years (Raikes & Love, 2002). Services provided under the EHS umbrella are accessed during home visits and/or at schools or childcare centers, and include parenting education, case management, community support, health advocacy, and child development interventions. Sessions occur twice per month for 90 min, and a child receives about 1,380 h of services through center-based programming. Broadly, EHS programs aim to enhance child development (e.g., early socio-emotional, cognitive, and language skills), health and safety, social support, financial wellbeing, and stress management for these at-risk families. Strengthening caregiver–child relationships is also a goal, and this is addressed through a focus on interpreting and sensitively responding to child needs, encouraging positive play interactions, and educating caregivers on child development (Roggman et al., 2009).

#### Evidence Base

Roggman et al. (2009) compared EHS to an unspecified control group using a RCT in a sample of 160 low-income mother–child dyads recruited during pregnancy or up to 10 months of postpartum. Child attachment security was assessed using the AQS (maternal report), when the children were 14 and 18 months old. Study retention was 85% and 80% at the 14- and 18-month assessments, respectively. Results showed the EHS to significantly predict attachment security at 18 months, independent of maternal depression and level of education. A potential study limitation, however, was the fact that the AQS was completed by mothers, so future studies using alternative approaches to the assessment of attachment are recommended.

### Watch, Wait, and Wonder (WWW)

#### Intervention Overview

Watch, Wait, and Wonder (WWW; Muir, 1992) is a psychodynamic approach to infant-caregiver psychotherapy delivered in 50-min weekly sessions for 8–20 weeks. The goals of WWW are to increase sensitive and responsive caregiving while addressing intergenerational attachment representations (Cohen et al., 1999). WWW differs from other psychodynamic caregiver–child therapies because it is infant-led; caregivers spend the first 30 min of each session interacting with their infants while letting the infant take the lead—with the aims of increasing caregiver insight and awareness into the child’s needs, and gaining the benefits of play therapy (Cohen et al., 1999; Muir, 1992). The final 20-min of therapy sessions involve a conversation between the therapist and caregiver about the observations and feelings that the caregiver had during the infant-led play interaction (Muir, 1992).

The program developers provide training in the WWW model (Watch Wait & Wonder, 2022). Training involves a 3-day experientially-based introductory workshop, followed 6–12 months later by an advanced consultation workshop or ongoing weekly or biweekly online sessions, both of which use a group case supervision process.

#### Evidence Base

Cohen et al. (1999) employed a RCT with a clinical sample of 67 mother–child dyads aged 10–30 months, randomly allocating them to receive WWW or psychodynamic psychotherapy (PPT). At the post-treatment assessment, 7 of the 34 of children in the WWW group (20.6%) had shifted from an insecure to secure attachment classification (using the SSP), compared to 1 of the 32 (3%) in the PPT group. Similarly, of the 34 children in the WWW group, 5 (14.7%) shifted to A or C, compared to 3 of the 32 (3%) in the PPT group. The authors reported that overall, children in the WWW group were significantly more likely to shift to a secure or organized attachment category (WWW 35.2%; PPT 12.5%) but they urged caution in interpretation of results as the changes were modest. In a subsequent study in which 87% of the same sample was followed up six months after the completion of treatment, Cohen et al. (2002) showed that while children from the WWW treatment group maintained their improvements in attachment security, there were no significant differences between them and the children in the PPT group with respect to attachment security, suggesting that both interventions brought about improvements but that the changes happened at different points. One of the major limitations of the study was the lack of a non-treated control group.

### UCLA Family Development Project

#### Intervention Overview

The UCLA Family Development Project is a manualized, relationship-based home-visiting intervention delivered by mental health professionals from late pregnancy through to the end of the child’s second year (Heinicke et al., 1999). Home visits take place on a weekly basis until the child’s first birthday, and then every second week in the child’s second year. Mother–infant groups are also made available to families throughout the program, and follow-up contact is provided to families in children’s third and fourth years. The primary goal of the UCLA Family Development Project is to provide mothers with a positive, trusting relationship experience with a home visitor. These relationships help mothers feel understood and supported, so they can work on problems. Continuity of care and shared pleasure in the child are key elements of the program (Heinicke et al., 2001).

#### Evidence Base

One RCT has examined attachment outcomes following the UCLA Family Development Project. In this study, Heinicke et al. (1999) evaluated attachment outcomes for 64 at-risk mothers recruited during pregnancy, 31 of whom were allocated to receive the UCLA Family Development Project intervention and 33 of whom were allocated to receive a control intervention (standard pediatric follow-up). Study retention was high (> 90%), and results showed that in the UCLA Family Development Project group, more than 3 times as many children showed a secure as opposed to insecure attachment classification, measured using the SSP when children with 14 months old. In contrast, the rates of secure versus insecure attachment in the control condition were virtually identical. They also found that disorganized attachment coupled with avoidance as a secondary classification was more common among the children in the control condition than it was for children in the intervention condition.

### Right from the Start

#### Intervention Overview

Right from the Start (RFTS; Niccols, 2008) is a group-based intervention that aims to enhance parental sensitivity and attachment security by improving parents’ understanding of attachment and the impact of parent and child temperament on parent–child interactions, and improving parents’ ability to correctly interpret child cues. RFTS is delivered by infant development specialists in weekly 2-h group sessions over an 8-week period. The sessions involve the presentation of video clips of confederate parents making exaggerated errors in common parent–child interaction situations, which are then discussed in both small (4–6 parents) and large (12–40 parents) groups. Parents practice skills in structured homework assignments between sessions and then discuss and support one another with home practice during the weekly sessions.

#### Evidence Base

Niccols (2008) conducted a RCT with 76 mothers who had infants ranging in age from 1 to 24 months. Participants were randomly allocated to receive RFTS (*n* = 48) or a control intervention (home visiting; *n* = 28). Attachment security was assessed (using the AQS) at post-treatment and at a 6-month follow-up. Of the 76 mothers who were randomized, 73 (96%) completed post-test measures, and 64 (84%) completed 6-month follow-up measures. Those who did not complete the study were less educated, but there were no other significant demographic differences between study completers and non-completers. Results showed no significant differences between groups in terms of the amount of change in attachment security. The authors did, however, report small but positive effect size differences for changes in infant attachment security for both groups. Study limitations included the lack of a non-treated control group, the use of a non-clinical sample at baseline, and the broad age range of the infants which meant that attachment outcomes were only available for a sub-group of the larger sample (those aged 9 months and over).

### Promoting First Relationships

#### Intervention Overview

Promoting First Relationships (PFR; Kelly et al., 2008) is a brief attachment-focused intervention aimed at increasing caregivers’ reflective functioning, ability to correctly interpret child cues, empathy for the child, and responsive caregiving. PFR is delivered in 60–75-min weekly home-based sessions, over a 10-week period, with sessions centering around presentation of written education materials (handouts) and parental reflections/discussions about video-taped caregiver–child interactions.

#### Evidence Base

Spieker et al. (2012) conducted an RCT with 210 caregiver–child dyads (child age *M* = 17.96, *SD* = 4.97 months), randomly allocating dyads to receive PFR or a comparison treatment (Early Education Support). Caregivers included birth parents (five fathers), kin, and foster carers; all study participants had experienced a court-ordered placement that resulted in the change of a primary caregiver within 7 weeks prior to study enrollment. Study authors assessed child attachment security using the Toddler Attachment Sort-45 (Kirkland et al., 2004) post-intervention (83% retention) and at a 6-month follow-up (61% retention). Results showed that while the PFR intervention was associated with significantly better attachment-related outcomes compared to the control group (e.g., sensitivity, understanding the child’s emotional needs), there were no significant differences in attachment security between intervention and control children. Caregiver heterogeneity within the sample made it difficult for the authors to be confident about the generalizability of results; this, combined with the small sample and thus lack of power, meant that sub-group analysis to examine differential impacts for different types of carers, was not possible.

### Moss’s Home-Visiting Intervention

#### Intervention Overview

Moss et al. (2011) described a brief, home-visiting intervention involving weekly 90-min visits from a trained clinician over an 8-week period. The intervention aims to increase caregiver-sensitive responding, improve caregiver support of child exploration, and address caregiver attachment representations. The home visit sessions comprise a 20-min discussion on a theme chosen by the parent, a 10–15-min parent–child interaction that is video-taped, and a 20-min feedback session in which the parent’s observations and feelings upon viewing the parent–child interaction video are discussed. The final 10–15 min of the session involve a ‘debrief’ during which the parent’s progress is highlighted, and they are encouraged to utilize the skills learned during the week.

#### Evidence Base

Moss et al. (2011) utilized an RCT to investigate the effectiveness of the home-visiting intervention compared to a non-treated control condition for families with histories of child maltreatment. The final sample included 67 parent–child dyads (4 father–child dyads and 63 mother–child dyads; child age range = 1.0–5.9, *M* = 3.35, *SD* = 1.38 years), representing 84% of the original randomized sample. Families in both groups continued to receive case management or child welfare services as usual. Child attachment was assessed at pre- and post-treatment using the SSP for infants aged 12–24 months and the Preschool Separation–Reunion Procedure (Cassidy et al., 1989) for infants aged 2–6 years of age. While attachment results were not reported separately for children aged under 2 and over 2 years, overall, results demonstrated that a significantly larger proportion of children in the home-visiting group showed pre- to post-treatment improvements in attachment security and organization. The authors highlighted that future studies should investigate differential outcomes for fathers and mothers, and test outcomes in different cultural groups.

### Lyons-Ruth’s Home-Visiting Intervention

#### Intervention Overview

Lyons‐Ruth et al. (1990) described a home-visitation program for families with infants at high social risk due to maternal depression, poverty, and caretaking inadequacy. The intervention comprised weekly sessions over a period of 9–18 months. The intervention goals were to (1) provide the parent with an experience of a trusting reliable relationship, (2) enhance the family’s ability to access resources to meet basic needs, (3) to promote positive and developmentally appropriate parent–infant interactions, and to encourage the parent’s role as an emotional source for the child, and (4) to decrease social isolation.

#### Evidence Base

Lyons‐Ruth et al. (1990) examined the effectiveness of the home-visiting intervention by comparing outcomes for a group of 31 mother–child dyads that received the home-visiting intervention (representing 89% of the original group of mothers who agreed to participate*;* child age *M* = 4.7 months), a control group of at-risk infants (*n* = 10), and a community group matched on social risk profile and paternal characteristics (*n* = 35). When child attachment was assessed at 18 months using the SSP, results showed that infants who had received the intervention had significantly lower rates of attachment insecurity and disorganization compared to the control group. Of the infants with depressed mothers, those who were untreated demonstrated more than twice the rate of insecure-disorganized behavior compared to the infants of depressed mothers in the intervention group. Results of the study are promising, but the study was limited by the non-randomized design.

### Minding the Baby

#### Intervention Overview

Minding the Baby is an attachment-based, home-visitation intervention designed to improve health, developmental, and relationship outcomes in at-risk young families who are having their first child (Slade et al., 2020). The program involves weekly home visits by a nurse and social worker throughout pregnancy and the child’s first year, followed by biweekly home visits for the child’s second year. During the home visits, the nurse and social worker provide the family with information on their child’s development, parent–child attachment, and parenting. Home visitors also attend to the basic needs of the family by supplying food, diapers, etc. The nurse provides the family with guidance on health, nutrition, and reproductive care. The social worker supports the family with approaches to addressing mental health concerns including complex trauma, depression, and anxiety. Training in the Minding the Baby intervention is provided through Yale University (Yale University, 2022).

#### Evidence Base

Slade et al. (2020) recruited 156 primiparous pregnant women and randomly allocated them to Minding the Baby (*n* = 77) or a control condition (perinatal and postnatal treatment as usual; *n* = 96). When the mother–child dyads were assessed mid-way through the intervention, at the age of 12 to 14 months (76% retention rate), children whose mothers received Minding the Baby were significantly more likely to be securely attached (as assessed using the SSP) and less likely to be disorganized than children in the control group. The results of this study are positive, but there were a high number of women who declined the intervention when invited (*n* = 101), and this is likely to have produced a biased sample. While the Minding the Baby intervention was delivered until the children were 24 months, at which point the researchers examined a range of other relevant outcome variables (parental reflective functioning, affective caregiving, and maternal mental health), infant attachment outcomes were only assessed at 12–14 months. This prevents any conclusions about the impact of Minding the Baby on attachment outcomes, when it is delivered throughout the child’s second year of life.

### Parent–Child Interaction Therapy-Toddlers

#### Intervention Overview

Parent–Child Interaction Therapy-Toddlers **(**PCIT-T) is an intervention designed for children aged 12 to 24 months with externalizing behavior problems and their parents (Girard et al., 2018). PCIT-T was developed as adaptation of the standard PCIT program for older children (Eyberg, 1988; Niec, 2018) and uses parent coaching during parent–child play sessions. The program comprises two phases, Child-Directed Interaction-Toddler (CDI-T), which aims to enhance children’s emotional regulation abilities by teaching parents to increase use of positive parenting skills, decrease negative parenting, increase parental sensitivity to the child’s emotional states, and actively support the child’s emotion regulation during times of distress. The CDI-T phase is followed by a Parent-Directed Interaction-Toddler (PDT-T) phase, which aims to enhance toddler listening skills through a developmentally tailored listening training procedure.

Training in the PCIT-T intervention is provided by the program developers (Parent–Child Interaction Therapy with Toddlers, 2022). For therapists who have already gained accreditation as a PCIT International certified therapist, PCIT-T training involves a 2-day workshop followed by 12 months of supervision/consultation calls through delivery of 2 completed PCIT-T cases. For non PCIT International certified therapists, a 5-day training workshop is provided followed by 12 months of supervision/consultation calls through delivery of 2 completed PCIT-T cases.

#### Evidence Base

Kohlhoff et al. (2020) evaluated child attachment outcomes following CDI-T phase of PCIT-T in a sample of mother–child dyads (child age range 15–24, *M* = 19.02, *SD* = 2.34 months) recruited through a community-based child behavior clinic for treatment of externalizing behaviors. All participants had initially taken part in an RCT study (comparing the CDI-T component of PCIT-T versus a waitlist control condition), following which those in the waitlist condition completed the CDI-T component of PCIT-T. Child attachment was assessed in 16 parent–child dyads who completed the SSP at baseline and then again 6 months later. In this one group of treatment completers, there were no statistically significant differences in the proportions of children who were insecure and disorganized from baseline to follow-up. The authors noted, however, that the odds ratios were in the expected direction, indicating lower odds of showing insecure and disorganized attachment at follow-up than at pre-treatment. The lack of a control group and the sample size were major limitations of this study (attachment outcomes assessed in only 16 of the 66 participants (24%) who were enrolled in the study). While the small sample size meant that the study was underpowered to find effects, the significant attrition rate is also likely to have led to significant sample bias.

### Results of Previous Meta-analyses

To date, there have been eight published meta-analyses of studies reporting attachment outcomes following attachment-based interventions for young children, and all of these have included, but were not limited to, toddlers (Bakermans-Kranenburg et al., 2003, 2005; Facompré et al., 2018; Letourneau et al., 2015; Van Ijzendoorn et al., 1995; Wright & Edginton, 2016; Wright et al., 2015, 2017). In the earliest of these studies, van Ijzendoorn and colleagues (1995) reviewed 16 studies investigating attachment-based interventions targeting maternal sensitivity and attachment security in infants. These authors conducted a meta-analysis of data from 12 studies. Results showed an effect size of *d* = 0.17 for improving the child–caregiver attachment relationship and suggested that shorter-term interventions and those with narrow foci (e.g., physical contact with infant) were more effective at improving attachment than longer-term interventions with broader foci (e.g., wraparound services).

Bakermans-Kranenburg et al. (2003) investigated 70 studies examining interventions aimed at enhancing attachment security among children younger than 54 months. Results showed aggregate effect sizes of *d* = 0.20 for improvement in infant attachment security. In addition, this meta-analysis provided evidence showing interventions which were brief (i.e., < 17 sessions), had a behavioral focus, and were conducted after the child was 6 months old, were associated with the greatest improvements in attachment security. Two years later, this same research group completed a meta-analysis of 15 studies reporting results of interventions aimed at preventing disorganized attachment styles (Bakermans-Kranenburg et al., 2005). Results again showed that the most successful interventions targeted children at least 6 months of age and focused mainly on increasing caregiver sensitivity as opposed to providing broader support or changing caregivers’ internal representations of attachment. Further, this meta-analysis indicated that interventions were more effective in samples with higher rates of attachment disorganization and in samples of “at-risk” children (e.g., irritable, premature) as opposed to those with “at-risk” caregivers (e.g., poor, socially isolated).

Letourneau et al. (2015) conducted a narrative systematic review and meta-analysis to examine the effectiveness of interventions targeting improvement of the caregiver–child attachment relationship in children young than 36 months by promoting maternal sensitivity and reflective functioning. Their sample included 10 studies, 7 of which were included in the meta-analysis. Results showed that most of the reviewed interventions led to significantly greater increases in attachment security compared to control conditions, with interventions targeting both maternal sensitivity and reflective functioning showing the greatest effects. Furthermore, the meta-analysis revealed that the overall effect size was significant and large (*d* = 1.53).

Wright and colleagues conducted a series of three reviews examining the evidence for attachment-based parenting interventions for children with severe behavior problems. In an initial study, Wright et al. (2015) showed greater effectiveness for treatments that targeted caregiver sensitivity and those that intervened in at-risk populations. Next, Wright and Edginton (2016) reviewed studies employing RCT designs and validated measures of attachment. Results showed greater improvements in attachment security and organization for intervention than control groups, but results between studies varied widely. Finally, Wright et al. (2017) reviewed attachment-based parenting interventions targeting children demonstrating or at-risk of developing disorganized attachment styles. The analysis led the authors to conclude that treatments aimed at improving caregiver sensitivity were most successful at decreasing disorganization but that few high-quality studies in this area existed. Overall, analyses revealed a medium effect size for decreases in disorganization across studies (*d* = 0.38).

Most recently, Facompré et al. (2018) conducted a meta-analysis to assess the effectiveness of interventions designed to prevent disorganized attachment in at-risk children. Their sample included 16 studies, all of which were (1) conducted between January 1989 and August 2016, (2) included a control condition, and (3) reported post-intervention rates of disorganized and organized attachment assessed using the SSP. Of the 16 studies, all but one was conducted with children aged less than 24 months at the start of the intervention. Results indicated that overall, compared to control conditions, interventions increased the rates of organized attachment (*d* = 0.35). Moderation analysis identified three indicators that were associated with the most optimal attachment outcomes, namely studies published after 2005 versus studies published before 2005, studies with maltreated versus non-maltreated samples, and studies with older versus younger children. In contrast to the results reported by Bakermans-Kranenburg et al. (2005), there were no statistically significant differences between the effectiveness of programs that focused on increasing parental sensitivity and programs that aimed to increase representations or support, however, the largest effect size was observed for programs that focused on maternal sensitivity (*d* = 0.51).

## Discussion

This review paper provides a narrative summary of available attachment-informed interventions for caregivers and toddlers (aged 12–24 months), an overview of evidence showing the ability of these interventions to bring changes in child attachment patterns, and a description of the available meta-analysis examining the ability of parenting interventions to change infant attachment patterns.

Overall, the programs with the strongest evidence base for changes in parent–child attachment when delivered in the early toddler years were CPP and ABC. The CPP intervention is supported by four separate RCTs and one follow-up study, including studies conducted by research teams who were uninvolved in the original development of the intervention (Cicchetti et al., 1999, 2006; Stronach et al., 2013; Toth et al., 2006). CPP was developed for high-risk parent–child dyads, so it is encouraging to see positive changes in attachment in high-risk samples. It must be noted, however, that of all the programs reviewed, CPP has one of the longest treatment durations and therefore is likely to be one of the most resource-intensive interventions. ABC was shown in two separate RCTs to be associated with positive child attachment outcomes (Bernard et al., 2012; Dozier et al., 2009), with 1 of these studies including a 9-year post-intervention follow-up (Zajac et al., 2020). These gains are impressive and encouraging given that ABC is a relatively brief intervention (10 weeks).

There was also evidence suggesting that various other interventions are also associated with positive attachment outcomes in toddlers (COS-HV4, STEEP, EHS, WWW, UCLA Family Development Project, Moss’s Home-Visiting Intervention, Lyons-Ruth’s Home-Visiting Intervention, Minding the Baby). However, while evidence for the great majority of these interventions came from RCTs, in most cases, evidence came from just one study, conducted by the program developers. It must also be noted that with the exception of two RCT studies (Dozier et al., 2009; Lieberman et al., 1991), attachment outcomes for the intervention and control group were assessed post-treatment only, rather than at both pre- and post-intervention. This means that individual changes in attachment over time were not examined, making it difficult to know whether the groups differed at baseline. For some interventions (e.g., COS-I, COS perinatal), available evidence about the interventions’ effect on attachment was positive but limited by the fact that it came from open-trial studies rather than RCTs. Future studies utilizing RCT designs should be conducted to test outcomes of these intervention programs further.

Finally, there were a couple of interventions for which the evidence of changes in toddler attachment was inconclusive (i.e., PCIT-T, RFTS). While it is possible that these programs are ineffective in addressing attachment issues in young children, such a conclusion is likely to be premature given that there has been so little research conducted on these programs to date. The only study conducted to assess attachment outcomes following PCIT-T, for example, used an open-trial design and had a small sample size. While results were non-significant, the study was underpowered, increasing the likelihood of a type II error (Kohlhoff et al., 2020).

Results from available meta-analyses suggest that targeting parenting sensitivity is the most effective way to change infant attachment security and organization. Other common features of effective interventions included being brief, having a behavioral focus, and being delivered after the child was 6 months old.

The current paper makes an important contribution to the literature because while previous reviews have examined the impact of attachment-based interventions on child attachment (Bakermans-Kranenburg et al., 2003, 2005; Facompré et al., 2018; Letourneau et al., 2015; Van Ijzendoorn et al., 1995; Wright & Edginton, 2016; Wright et al., 2015, 2017), these have included children from a wide range of ages rather than focusing specifically on interventions delivered in the period of early toddlerhood. In addition, in most cases, the focus of previous reviews was on meta-analysis rather than narrative review of available programs. The strengths of this paper must be considered, however, alongside an acknowledgment of its limitations. The review was not systematic in nature, and while every effort to uncover relevant studies was taken, it is possible that some studies may have been missed. Furthermore, only English language studies were reviewed due to the costs associated with translation, reducing the generalizability of findings.

In sum, this paper describes the attachment-based parenting interventions that have been developed to improve the quality of caregiver–child attachment relationships, and that have been applied to the early toddler age group. There was evidence to suggest that a number of interventions were associated with changes in child attachment, with CPP and ABC emerging as the interventions with the strongest evidence bases. For most of the other interventions, however, evidence came from just a single research study, and in some cases the studies lacked control groups. In order for clinicians to make informed decisions about the interventions they use with parents and toddlers, it is vital that more studies be conducted to test each intervention, and that future studies should use RCT designs, in a range of settings and clinical and cultural groups, and with longitudinal follow-ups.

## Data Availability

There are no specific data files associated with this manuscript.
